# C-reactive protein and fatigue after subarachnoid haemorrhage

**DOI:** 10.1016/j.bbih.2025.101046

**Published:** 2025-06-30

**Authors:** Sean Daniel, Ruihua Hou, Ian Galea, Diederik Bulters

**Affiliations:** a Department of Neurosurgery, Wessex Neurological Centre, Southampton General Hospital, Southampton, UK; b Clinical and Experimental Sciences, Faculty of Medicine, University of Southampton, Southampton, UK; c Clinical Neurosciences, Clinical and Experimental Sciences, Faculty of Medicine, University of Southampton, Southampton, UK

## Abstract

**Background:**

Subarachnoid haemorrhage (SAH) is a severe type of intracranial bleed that causes significant morbidity. One of the most overlooked yet commonly reported symptom is persistent fatigue. Fatigue in the general population has been associated with inflammation and elevated C-reactive protein (CRP). Since inflammation and raised CRP are observed post-SAH, we hypothesized that CRP is associated with fatigue severity after SAH.

**Methods:**

Data from 95 patients (26 males, 69 females; mean age 56 years) previously recruited to the SAS trial, a prospective randomised controlled trial (2016–2019) investigating the efficacy of sulforaphane after SAH, were analyzed. In this study CRP was measured on days 1, 7, and 28 post-SAH, and fatigue severity assessed using the Short-Form Health Survey (SF-36) on days 28, 90, and 180. Multivariable regression was conducted controlling for confounders, including age, gender, initial volume of blood on CT and World Federation of Neurological Surgeons (WFNS) grade.

**Results:**

There was a robust association between the severity of fatigue at day 28 and CRP levels at baseline (OR = -2.67 (−1.23 to −0.18), p = 0.001) and CRP on day 28 (OR = -2.56 (−1.01 to −0.12), p = 0.013), even after controlling for confounders including blood volume and WFNS. There was no suggestion of an association between day 7 CRP and fatigue on day 28 (OR = -1.07 (−0.82 to 0.25), p = 0.287). There were no associations with any other fatigue timepoints.

**Conclusion:**

CRP and fatigue in SAH patients are associated. The timings of the associations of baseline and day 28 CRP (but not day 7) with day 28 fatigue, and their independence from bleed severity suggest that fatigue is related partly to the magnitude of the initial response to the SAH and partly due to the degree of ongoing response at day 28 but not due to other events occurring in between. The lack of association of early CRP with fatigue beyond day 28 suggests that later fatigue is not driven by the initial CRP-related response to SAH. Further studies are needed to examine later CRP and the determinants of persistent fatigue.

## 1 Introduction

Subarachnoid haemorrhage (SAH) is a type of stroke characterized by bleeding within the subarachnoid space most commonly caused by rupture of an aneurysm on or near the circle of Willis. Although SAH accounts for only 5 % of all strokes, it is responsible for more morbidity and mortality than any other stroke type. Approximately 30 % die, and even survivors deemed to have made a good recovery suffer a host of symptoms including cognitive, emotional and psychological problems. Among the most prevalent and debilitating sequelae are mental and physical fatigue (Visser-Meily et al., 2009), affecting as many as 70 % of patients up to 7 years after the haemorrhage (Western and Sorteberg, 2020), drastically reducing Quality of life (QoL) (Visser-Meily et al., 2009) and hindering return to normal life and work long after resolution of the acute event (Gaastra et al., 2022). So far, the biological mechanisms underlying fatigue after SAH remain unclear.

Fatigue has been more extensively studied in the general population. Fatigue is one of the most common symptoms reported in clinical consultations (Sharpe and Wilks, 2002). Although it can manifest transiently in over 50 % of the healthy population, some patients may develop chronic fatigue, in which symptoms can persist consistently for over 6 months after initial presentation (Afari and Buchwald, 2003). It is also recognized in other conditions like diabetes, cardiovascular disease and cancer (Bower, 2014; Markanday, 2015). There is growing evidence that inflammation may contribute to the development of fatigue. Several longitudinal studies analyzing the biomedical parameters of healthy volunteers in large databanks have suggested that there is a direct link between inflammation and fatigue, and that C-reactive protein (CRP) was a good predictor of future self-reported fatigue (Cho and Kivimaki, 2013; Hughes and Kumari, 2018; Milaneschi et al., 2021; Sharpley et al., 2019). This is known as the cytokine-induced inflammatory-mediated fatigue hypothesis, which suggests that increased cytokines in the central nervous system (CNS) may alter the baseline functioning of the dopaminergic pathways in the brain, ultimately blunting cognition, motivation, and effortful behaviour (Western and Sorteberg, 2020; Western et al., 2021). Although theoretical, this suggestion is supported by experimental data highlighting that cytokines can disrupt dopamine processing by targeting the basal ganglia (Felger and Miller, 2012.

SAH is characterized by both a strong systemic and CNS-specific inflammatory response (Markanday, 2015). The presence of arterial blood in the subarachnoid space, in particular metabolites of haemoglobin, activates local microglia and endothelial cells. Resident immunocompetent cells in the CNS play a crucial role in inflammation (Chen et al., 2014). They release proinflammatory cytokines and encourage neutrophil and macrophage migration within the brain (Alghamdi et al., 2021). As a result, there is upregulated endothelial cell apoptosis, glial cell necrosis and nervous tissue damage in the first 48 h after the event (Chai et al., 2023; Chen et al., 2014). Once the local inflammatory reaction is underway, there is overspilling of cytokines into the bloodstream, thus perpetuating a systemic response to the bleed (Chai et al., 2023; Savarraj et al., 2017; Woodburn et al., 2021). Interleukin-6 (IL-6) is one of the most strongly upregulated cytokines. Of note, it directly stimulates CRP release and activation (Chen et al., 2014; Markanday, 2015).

CRP is an acute-phase reactant intricately involved in orchestrating the inflammatory response, wielding a dual role in promoting both pro-inflammatory and anti-inflammatory processes (Markanday, 2015). CRP is produced in the liver and serves as a sentinel, enhancing the immune system's ability to detect foreign pathogens and apoptotic or necrotic cells through its phosphocholine-binding properties. Moreover, it amplifies the complement system, a vital component of the immune cascade, which perpetuates inflammation if the initial threat persists (Chen et al., 2014; Markanday, 2015). Clinically, CRP levels serve as a dependable biomarker of inflammation severity as production increases proportionally to the extent of the stimulus and are widely used to assess the gravity of acute medical conditions. CRP is acutely raised after SAH and has been shown to be predictive of outcome, typically assessed by mortality, functional deficits and physical and psychological symptoms (Gaastra et al., 2021; Hong et al., 2014; Juvela et al., 2012; Liu et al., 2020; Romero et al., 2012). There are no studies of CRP and fatigue after SAH, however. The only available literature comes from ischaemic stroke and clinical outcomes other than fatigue (Alghamdi et al., 2021; Juvela et al., 2012; Liu et al., 2020), and there is no data beyond day 14 after the event.

Therefore, the overall aim of this study was to investigate whether inflammation is associated with the development of fatigue in patients after SAH. Specifically, we set out to examine the associations between CRP and fatigue after SAH at critical timepoints. For CRP these timepoints corresponded to early brain injury (day 1), delayed brain injury and vasospasm (day 7) and following resolution of the acute inflammatory response (day 28). For fatigue, these timepoints were short (day 28), medium (day 90) and long-term (day 180) fatigue post SAH. Our hypothesis was that if post-SAH fatigue is mediated by active and concurrent inflammation, the CRP and fatigue on day 28 would be associated, while there would be no or weak association at other timepoints. However, if post-SAH fatigue was due to the magnitude of the injury caused by the SAH (or the injury caused by the early transient inflammation after SAH), then CRP at the time of early or delayed brain injury would be more strongly associated with fatigue.

## 2 Methods

### 2.1 Participants

This study was an exploratory secondary analysis of data obtained during a multicentre double-blind controlled, parallel-group study. The original SAS study approached and consented 105 patients aged 18–80 years who had suffered a Fisher grade 3 or 4 SAH within the last 48 h. They were recruited in three specialist tertiary UK centres in Southampton, London and Edinburgh between April 2016 and September 2019 and followed up for 6 months after the SAH event. The primary objective of the original study was to assess the safety, pharmacokinetics and efficacy of SFX-01 in patients with SAH. In the main study no effect of sulforaphane was seen on any blood parameters or any clinical outcomes. The full study details including blood sample collection and ascertainment of outcomes are available in the published protocol (Zolnourian et al., 2020) and manuscript (Zolnourian et al., 2024).

The trial protocol met the international criteria for Good Clinical Practice Guidelines and was approved by relevant independent ethics committee (National research ethics service – Southern central Hampshire A) and Medicinal Health Care Authority (MHRA) and was registered (NCT02614742). Consolidated Standards of Reporting Trials (CONSORT) were followed as part of reporting guidelines.

The key inclusion criteria for the original SAS study were: 1) Radiological evidence of spontaneous aneurysmal SAH, 2) Fisher grade 3 or 4 on CT imaging, 3) Age between 18 and 80 years, 4) Randomization and initiation of treatment within 48 h of SAH.

For inclusion in the current study, we required: 1) CRP levels to be available from at least two timepoints, and 2) At least two of their three assessments of fatigue to have been completed.

### 2.2 Outcome measures

Measures of inflammation: Blood samples, including CRP levels, recorded in mg/L, were collected on Day 1, Day 7, and Day 28 after SAH in the SAS study and assayed by the local clinical biochemistry service at University Hospital Southampton using a.

Particle-enhanced Immunoturbidimetry assay performed on an automated Beckman Coulter AU680 analyser using the accompanying CRP latex reagent. These three timepoints correspond to different stages of the recovery post-SAH and were selected as indicators of inflammation at the time of early brain injury, delayed brain injury (including vasospasm), and resolution of SAH. There were no blood samples available beyond 28 days after SAH.

Measures of bleed severity: The clinical severity of the bleed was measured with the World Federation of Neurological Surgeons (WFNS) grade obtained at the time of randomization. The radiological severity was graded by Fisher scores. However, due to the severe limitations of Fisher scoring all CT scans were analyzed for blood volume, quantified in milliliters; this was used for radiological severity instead.

Measures of fatigue: Short Form-36 (SF-36) questionnaires were available from day 28, 90 and 180 after SAH. The questionnaire details 8 domains to measure the health-related quality of life of patients through multiple-choice questions. We extracted questions quantifying daily energy as a measure of fatigue, with a reliability of ⍺ = 0.86. The scores were then aggregated into a Likert scale ranging from 0 to 5 and modified to ensure unidirectionality, as validated by RAND (Ware and Sherbourne, 1992), where a higher score reflects a more favourable health state and there is a maximum of 20 points that can be achieved per timepoint. The questions were: 1) Did you feel full of pep?, 2) Did you have a lot of energy?, 3) Did you feel worn out? 4) Did you feel tired?

**Fig. 1:**
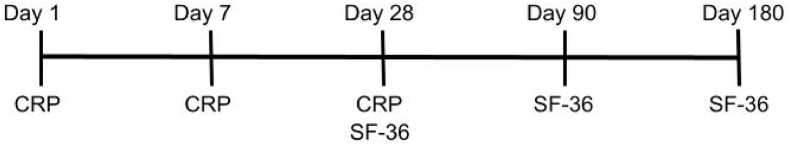
Linear timeline of datapoint collection from baseline to day 180, illustrating C-reactive protein levels and fatigue scores (SF36).

The timeline of CRP and fatigue measurements is presented below in Fig. 1.

### 2.3 Analysis

We performed linear regression of the three CRP measurements to predict fatigue at each timepoint. Both unadjusted and adjusted analyses were performed to account for any potential covariates, including patient characteristics (age, gender, body mass index), lifestyle choices (smoking, drinking and illicit drug use) and bleed severity (volume of blood on CT scan (Galea et al., 2022), quantified in milliliters and WFNS grade and Fisher score). Additionally, pharmacological management with SFX-01 or placebo was included as a covariate.

Univariable regression analysis was performed to assess the predictive power of the control variables against the three fatigue timepoints. Those that remained at the end of the analysis and demonstrated significance of p < 0.05 at any of the three timepoints were considered for inclusion for multivariate analyses at all timepoints. Therefore, the same covariates were used in all multivariate models simplifying comparison between timepoints.

In cases missing one fatigue measurement, imputation was performed using the “value carried forward” or where not possible “value carried backward” approach. This method ensured that missing data points were consistent with the answers given to other questions within the same sub-domain. Residuals were tested for skew and heteroscedasticity and data log transformed if indicated. All data analysis was performed using IBM SPSS 29.0.2.0 (20) Statistics Subscription. STROBE guidelines were followed throughout for this analysis.

## 3 Results

### 3.1 Study population

**Fig. 2:**
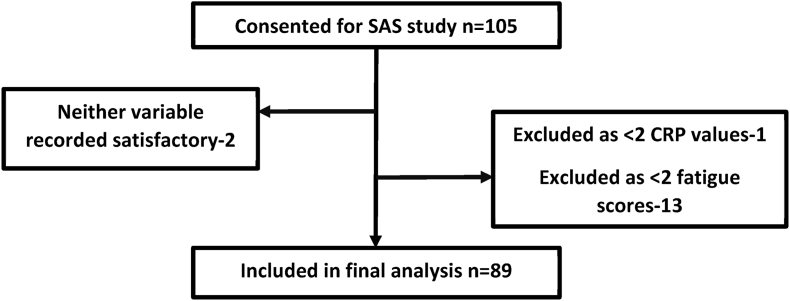
Flow diagram demonstrating selection of the patient cohort.

Of the 105 patients who consented to SAS, 89 met the inclusion criteria for this study and were therefore included in the final analysis, as per Fig. 2. The patient demographics are shown in Table 1.

**Table 1:** – Demographics of eligible patients (mean ± SD).

Sample size		N = 89
Age (years)		56 ± 12.0
**Gender**	Male	23 (25.8 %)
	Female	66 (74.2 %)
**Blood volume on CT (ml)**		25.6 ± 18.1
**WFNS**	**I**	39 (34.2 %)
	**II**	12 (10.5 %)
	**III**	6 (5.3 %)
	**IV**	26 (22.8 %)
	**V**	3 (2.6 %)
**CRP (mg/L)**	**Day 1**	31.4 ± 40.6
	**Day 7**	56.0 ± 60.5
	**Day 28**	13.7 ± 27.0
**Fatigue**	**Day 28**	1.7 ± 1.1
	**Day 90**	2.2 ± 1.2
	**Day 180**	2.4 ± 1.3

### 3.2 Association between clinical characteristics and fatigue

Univariable and multivariable regression of available covariates on fatigue showed that age, gender and initial SAH blood clot volume independently predicted fatigue at least once within the tested timepoint and were taken forward as covariates in the main analysis. Conversely, body mass index, site of aneurysm, use of SFX-01, Fisher score, WFNS, use of recreational drugs or psychoactive medications were not independently predictive of any of the three timepoints and were thus excluded from the final analysis.

### 3.3 Associations between CRP and fatigue

**Fig. 3:**
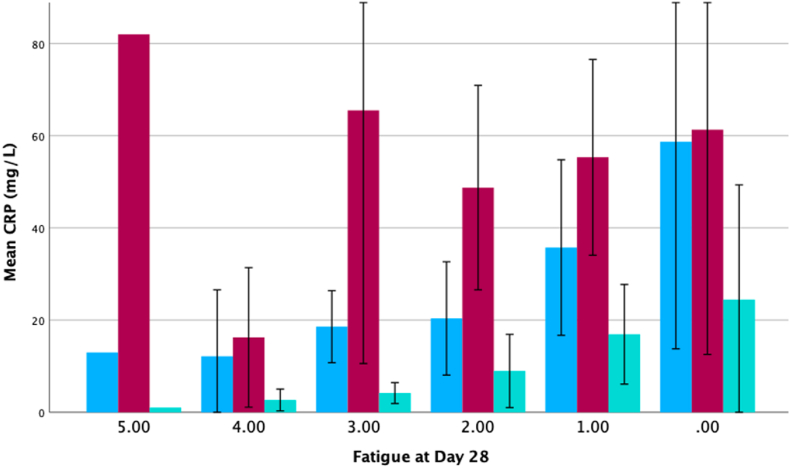
Mean CRP and fatigue on Day 28. Fatigue scores out of 5 derived from SF-36 as validated by RAND, where a lower score suggests more severe fatigue. Note the reversed x-axis, with scores decreasing from left to right since lower scores indicate higher levels of fatigue. Note the incremental increase in CRP with fatigue on day 28 in blue (CRP day 1) and turquoise (CRP day 28) but no trend seen in red (CRP day 7). No similar relationships of CRP with fatigue were seen at day 90 or day 180 (see Fig. 4 and 5 in Supplement). (For interpretation of the references to colour in this figure legend, the reader is referred to the Web version of this article.)

The relationship between CRP levels and fatigue at different timepoints is displayed in Fig. 3. There was an incremental increase in CRP on day 1 and day 28 with each step increase in fatigue on day 28. No such relationship was observed with CRP on day 7, or with any CRP measurements with fatigue on day 90 or 180.

Unadjusted univariable and adjusted multivariable linear regression was performed with log transformed CRP levels at day 1, day 7 and day 28 to predict fatigue. Any variable significantly associated on univariable regression at any time (age, gender and initial SAH blood) was included as a covariate as described above. The results of the regression are presented in Table 2.

**Table 2:** Associations between CRP and fatigue at different timepoints. ∗∗p < 0.01, ∗p < 0.05, ‘p < 0.1

		UNADJUSTED		ADJUSTED	
		Effect (β)	p-value	Effect (β)	p-value
**Fatigue Day 28**	**CRP D1**	−2.65 (−0.98–−0.14)	0.009*∗∗*	−2.67 (−1.23–−0.18)	0.001*∗∗*
	**CRP D7**	−1.30 (−0.76–0.16)	0.196	−1.07 (−0.82–0.25)	0.287
	**CRP D28**	−3.15 (−1.05–−0.24)	0.002*∗∗*	−2.56 (−1.01–−0.12)	0.013 ∗
**Fatigue Day 90**	**CRP D1**	−1.78 (−0.91–0.51)	0.079’	−1.27 (-1.04–0.23)	0.208
	**CRP D7**	−1.68 (−0.94–0.07)	0.096′	−1.22 (−0.99–0.24)	0.225
	**CRP D28**	−2.40 (−1.09–−0.10)	0.019∗	−1.89 (−0.99–0.03)	0.062′
**Fatigue Day 180**	**CRP D1**	−0.54 (−0.66–0.38)	0.587	0.57 (−0.48–0.86)	0.569
	**CRP D7**	−1.60 (−0.99–0.11)	0.114	−0.75 (−0.89–0.41)	0.457
	**CRP D28**	−1.14 (−0.95–0.17)	0.171	−0.66 (−0.73–0.37)	0.515

## 4 Discussion

In this study we investigated the association between CRP and fatigue severity across different timepoints following SAH. We demonstrate that CRP and fatigue were associated, independent of bleed severity. Fatigue on day 28 was predicted by CRP measurements on days 1 and 28 but not day 7. Moreover fatigue on day 90 and 180 were not predicted by any CRP measurement on days 1, 7 and 28. Both CRP at time of early brain injury (reflected by the day 1 assay) and on day 28 were associated with fatigue, but not CRP at time of delayed brain injury or complications related to hospitalisation (on day 7), contrary to previous studies (de Rooij et al., 2013; Fountas et al., 2009; Hwang et al., 2013). The effect sizes of day 1 and day 28 CRP on fatigue on day 28 were similar. We conclude that the response (pro-inflammatory or otherwise) associated with early brain injury (as indicated by day 1 CRP) was prolonged such that (a) it was related to day 28 fatigue and (b) this relationship persisted at one month. However on day 7, any effect of CRP on day 28 fatigue was eclipsed by major events such as delayed cerebral ischaemia, post-surgical trauma, instrumentation, metabolic upset or infection, which are likely to be short-lived and reversible.

### 4.1 Early CRP as predictor of later fatigue

There was a strong relationship (P = 0.001) between CRP on day 1 and fatigue on day 28, which indicates that baseline CRP response is a good predictor of fatigue a month later. Initially we hypothesized that this relationship was dependent on the severity of the bleed – which would yield a higher CRP spike and worse overall outcome (Hong et al., 2014; Juvela et al., 2012; Juvela and Siironen, 2012; Romero et al., 2012) – and therefore predict fatigue. But as the association was independent of the volume of blood on CT and clinical WFNS score, the predictive power of CRP cannot be attributed to the extent of the initial injury alone. The host response to early brain injury, which can vary widely between individuals, would explain this, however. More specific inflammatory mediators, such as cytokines (Fassbender et al., 2001), chemokines (Mellergard et al., 2010) and cellular adhesion molecules (Kubo et al., 2008), might be more closely associated with early brain injury and could be employed alongside CRP in future studies to better understand the relationship between inflammation and fatigue. CSF (or brain interstitial fluid) rather than blood measurements would also be invaluable, albeit challenging to obtain samples.

### 4.2 Delayed brain injury not associated with fatigue

Although CRP was highest on day 7, there was no relationship between CRP at day 7 and fatigue at any timepoint. This could simply be because CRP rises from delayed brain injury are not associated with levels of fatigue. However, although day 7 CRP levels may reflect delayed brain injury, this was not necessarily the case. Although studies suggesting that early CRP might be a marker for vasospasm after SAH (Fountas et al., 2009; Hwang et al., 2013; de Rooij et al., 2013), there is no established causative evidence and later CRP rises may be more multifactorial. It is likely that day 7 will also be influenced by short-lived stimuli independent of SAH, such as concomitant systemic inflammation associated with treatments, instrumentation, metabolic upset or infection. Being short-lived, such factors would not necessarily influence day 28 fatigue. Either way, day 7 CRP's poor predictive power suggests that delayed brain injury is not a driver for long-term fatigue.

### 4.3 Association between concurrent CRP and fatigue

Our study supports the hypothesis that fatigue severity at one month after SAH directly reflects concurrent CRP levels, as we found a clear relationship (p = 0.013) between CRP and fatigue on day 28, even after controlling for confounding factors. It is therefore possible that in some patients the initial response persists, predisposing to fatigue long after the bleed has subsided; this would be analogous to a study in healthy volunteers which supported a linear relationship between persistently elevated CRP and fatigue severity (Cho, 2009a, Cho, 2009b). Although association does not prove causality, the data from this study is in keeping with the current understanding that CRP-associated fatigue is influenced by prolonged rises in CRP, rather than short-lived spikes (Cho and Kivimaki, 2013; Cho, 2009a, Cho, 2009b). By day 28, CRP levels would not be expected to be influenced by short-term complications occurring around day 7.

### 4.4 Clinical implications

Recognizing fatigue as a well-known complication of SAH is important. Day 1 CRP levels provide a means for proactive and inexpensive screening and intervention. Early identification and treatment of the underlying inflammatory response, coupled with psychological counselling, could enhance well-being and contribute to social stability during the recovery phase and ultimately improve long-term outcomes after SAH.

Previous studies have already indicated that post-SAH fatigue correlates with long-term unemployment (Gaastra et al., 2022), reduced quality of life (Western et al., 2021) and other health and social consequences, emphasizing the need for targeted interventions. This study suggests that inflammation may be an important driver of fatigue but does not prove it. In that context, it will be particularly interesting to see the results of the secondary analysis from the recently completed SC IL1-RA trial (NCT03249207) of the interleukin 1 receptor antagonist, Kineret as a treatment after SAH, which included fatigue assessments.

### 4.5 Limitations and future directions

It is worth noting certain limitations in this study. Firstly, the SF-36 assessment of fatigue might not have been exhaustive enough to include all manifestations of fatigue, such as mental or physical subtypes. Other tools might prove to be more sensitive and specific, such as the Fatigue Severity Scale. Secondly, the absence of CRP measurements after 28 days was a significant limitation imposed by the data available. Thirdly, other factors known to impact fatigue should be considered, such as exercise and diet, psychological support, and even the role of personality type and coping mechanisms in long-term fatigue (Cho, 2009a, Cho, 2009b; Gaastra et al., 2022). This will help the development of a more personalized approach to support patients experiencing fatigue after SAH. Fourthly, the sample size was modest and skewed towards those with more severe SAH. Lastly, CRP is known to be a generic indicator of inflammation that is not produced by brain tissue. It can be easily modulated by several other local and systemic processes, which makes it prone to fluctuations and difficult to control long-term. Therefore, it would be worthwhile to investigate more direct measures of CNS inflammation such as CSF cytokines in future studies of fatigue after SAH. It is possible that CRP is not acting as a biomarker of inflammation, but something else, for instance the hepatic acute phase response to early brain injury (Bandyopadhyay et al., 2023; Wilcockson et al., 2002).

## 5 Conclusion

This study highlights the association between fatigue severity and CRP levels in patients after SAH, which is independent of bleed severity. The findings indicate potential mechanisms underlying fatigue after SAH. Since early CRP predicted fatigue 28 days later, this may suggest a causal relationship, but this remains to be proven. Insight from this study may inform novel intervention approaches to improve fatigue after SAH.

## CRediT authorship contribution statement

**Sean Daniel:** Writing – review & editing, Writing – original draft, Project administration, Methodology, Investigation, Formal analysis, Data curation. **Ruihua Hou:** Writing – original draft, Supervision, Methodology, Formal analysis, Conceptualization. **Ian Galea:** Writing – review & editing, Validation, Supervision, Methodology, Data curation, Conceptualization. **Diederik Bulters:** Writing – review & editing, Writing – original draft, Supervision, Methodology, Investigation, Formal analysis, Data curation, Conceptualization.

## Informed consent statement

Written informed consent was obtained from all participants or their legal representatives.

## Funding

I.G. was supported by a Medical Research Council (MRC) grant (MC_PC_17177). The SFX-01 study was funded by Evgen Pharma.

## Declaration of competing interest

The authors declare no conflict of interest.

## Data Availability

The data presented in this study are available on request subject to ethical, funder and institutional approvals.

## References

[bib1] Afari N., Buchwald D. (2003). Chronic fatigue syndrome: a review. Am. J. Psychiatr..

[bib2] Alghamdi I. (2021). Prevalence of fatigue after stroke: a systematic review and meta-analysis. European Stroke J..

[bib4] Bandyopadhyay S. (2023). The haptoglobin response after aneurysmal subarachnoid haemorrhage. Int. J. Mol. Sci..

[bib5] Bower J. (2014). Cancer-related fatigue -- Mechanisms, risk factors and treatments. Nat. Rev. Clin. Oncol..

[bib7] Chai C., Ho U., Kuo L. (2023). Systemic inflammation after aneurysmal subarachnoid haemorrhage. Int. J. Mol. Sci..

[bib8] Chen S. (2014). Controversies and evolving new mechanisms in subarachnoid haemorrhage. Prog. Neurobiol..

[bib9] Cho H., Kivimaki M.e. a. (2013). Association of C-reactive protein and interleukin-6 with new-onset fatigue in the Whitehall II prospective cohort study. Psychol. Med..

[bib10] Cho H. (2009). Prospective association between C-reactive protein and fatigue in the coronary artery risk development in yound adults study. Biol. Psychiatry.

[bib11] Cho J. (2009). Prospective association between C-reactive protein and fatigue in the coronary artery risk development in young adult study. Biol. Psychiatry.

[bib12] de Rooij N., Rinkel G., Dankbaar J., Frijns C. (2013). Delayed cerebral ischaemia after subarachnoid haemorrhage: a systematic review of clinical, laboratory and radiological predictors. Stroke.

[bib13] Fassbender K., Hodapp B., Rossol S.e. a. (2001). Inflammatory cytokines in subarachnoid haemorrhage; association with abnormal blood flow velocities in basal cerebral arteries. J. Neurol. Neurosurg. Psychiatr..

[bib14] Felger J., Miller A. (2012). Cytokine effects on the basal ganglia and dopamine function: the subcortical source of inflammatory malaise. Front. Neuroendocrinol..

[bib15] Fountas K., Tasiou A., Kapsalaki E. (2009). Serum and cerebrospinal fluid C-reactive protein levels as predictors of vasospasm in aneurysmal subarachnoid haemorrhage: clinical article. Neurosurg. Focus.

[bib16] Gaastra B. (2021). CRP (C-reactive protein) in outcome prediction after subarachnoid haemorrhage and the role of machine learning. Stroke.

[bib17] Gaastra B., Carmichael H., Galea I., Bulters D. (2022). Long-term fatigue following aneurysmal subarachnoid haemorrhage and the impact on employment. Eur. J. Neurol..

[bib18] Galea I. (2022). Iron deposition in the brain after aneurysmal subarachnoid haemorrhage. Stroke.

[bib19] Hong C. (2014). Biomarkers as outcome predictors in subarachnoid haemorrhage - a systematic review. Biomarkers.

[bib20] Hughes A., Kumari M. (2018). Age modification of the relationship between C-reactive protein and fatigue: findings from Understanding Society (UKHLS). Psychol. Med..

[bib21] Hwang S. (2013). Significance of C-reactive protein and transcranial Doppler in cerebral vasospasm following aneurysmal subarachnoid hemorrhage. J. Korean Neurosurg. Soc..

[bib22] Juvela S., Kuhmonen J., Siironen J. (2012). C-reactive protein as a predictor for poor outcome after aneurysmal subarachnoid haemorrhage. Acta Neurochir..

[bib23] Juvela S., Siironen J. (2012). Early cerebral infarction as a risk factor for poor outcome after aneurysmal subarachnoid haemorrhage. Eur. J. Neurol..

[bib24] Kubo Y. (2008). Serum inflammatory adhesion molecules and high-sensitivity C-reactive protein correlates with delayed ischemic neurologic deficits after subarachnoid hemorrhage. Surg. Neurol..

[bib25] Liu X. (2020). Elevated plasma high-sensitivity C-reactive protein at admission predicts the occurrence of post-stroke fatigue at 6 months after ischaemic stroke. Eur. J. Neurol..

[bib26] Markanday A. (2015). Acute phase reactants in infection: evidence-based review and a guide for clinicians. Open Forum Infect. Dis..

[bib27] Mellergard P., Sjogren F., Hillman J. (2010). Release of VEGF and FGF in the extracellular space following severe subarachnoidal haemorrhage or traumatic head injury in humans. Br. J. Neurosurg..

[bib28] Milaneschi Y. (2021). Association of inflamation with depression and anxiety: evidence for symptom-specific and potential causality from UK Biobank and NESDA cohorts. Mol. Psychiatr..

[bib29] Romero F., de Bertolini E., Figueiredo E., Teixeira M. (2012). Serum C-reactive protein levels predict neurological outcome after aneurysmal subarachnoid haemorrhage. Arq. Neuropsichiatr..

[bib30] Savarraj J. (2017). Systematic model of peripheral inflammation after subarachnoid haemorrhage. Neurology.

[bib31] Sharpe M., Wilks D. (2002). Fatigue. Br. Med. J..

[bib32] Sharpley C. (2019). The association between cortisol:C-reactive protein ratio and depressive fatigue is a function of CRP rather than cortisol. Neuropsychiatric Dis. Treat..

[bib34] Visser-Meily J. (2009). Long-term health related quality of life after aneurysmal subarachnoid haemorrhage; relationship with psychological symptoms and personality characteristics. Stroke.

[bib35] Ware J., Sherbourne C. (1992). The MOS 36-Item Short-Form Health Survey (SF-36): I. Conceptual framework and item selection. Med. Care.

[bib36] Western E. (2021). Fatigue after aneurysmal Subarachnoid haemorrhage: clinical characteristics and associated factors with patients with good outcome. Front. Behav. Neurosci..

[bib37] Western E., Sorteberg A.e. a. (2020). Prevalence and predictors of fatigue after aneurysmal subarachnoid haemorrhage. Acta Neurochir..

[bib38] Wilcockson D., Campbell S., Anthony D., Perry H. (2002). The systemic and local acute phase response following acute brain injury. J. Cerebr. Blood Flow Metabol..

[bib39] Woodburn S., J B., Wohleb E. (2021). The semantics of microglia activation: neuroinflammation, homeostasis and stress. J. Neuroinflammation.

[bib40] Zolnourian A., Franklin S., Galea I., Bulters D. (2020). Study protocol for SFX-01 after subarachnoid haemorrhage (SAS): a multicentre randomised double-blinded, placebo controlled trial. BMJ Open.

[bib41] Zolnourian A. (2024). A randomised controlled trial of SFX-01 after Subarachnoid haemorrhage - the SAS Study. Transl. Stroke Res..

