# Supplementary material for: C-reactive protein and fatigue after subarachnoid haemorrhage
Source: Brain Behav Immun Health. 2025 Jun 30;48:101046. doi: 10.1016/j.bbih.2025.101046 (PMC12312114; doi:10.1016/j.bbih.2025.101046)
Supplement: Multimedia component 1 [file mmc1.docx]

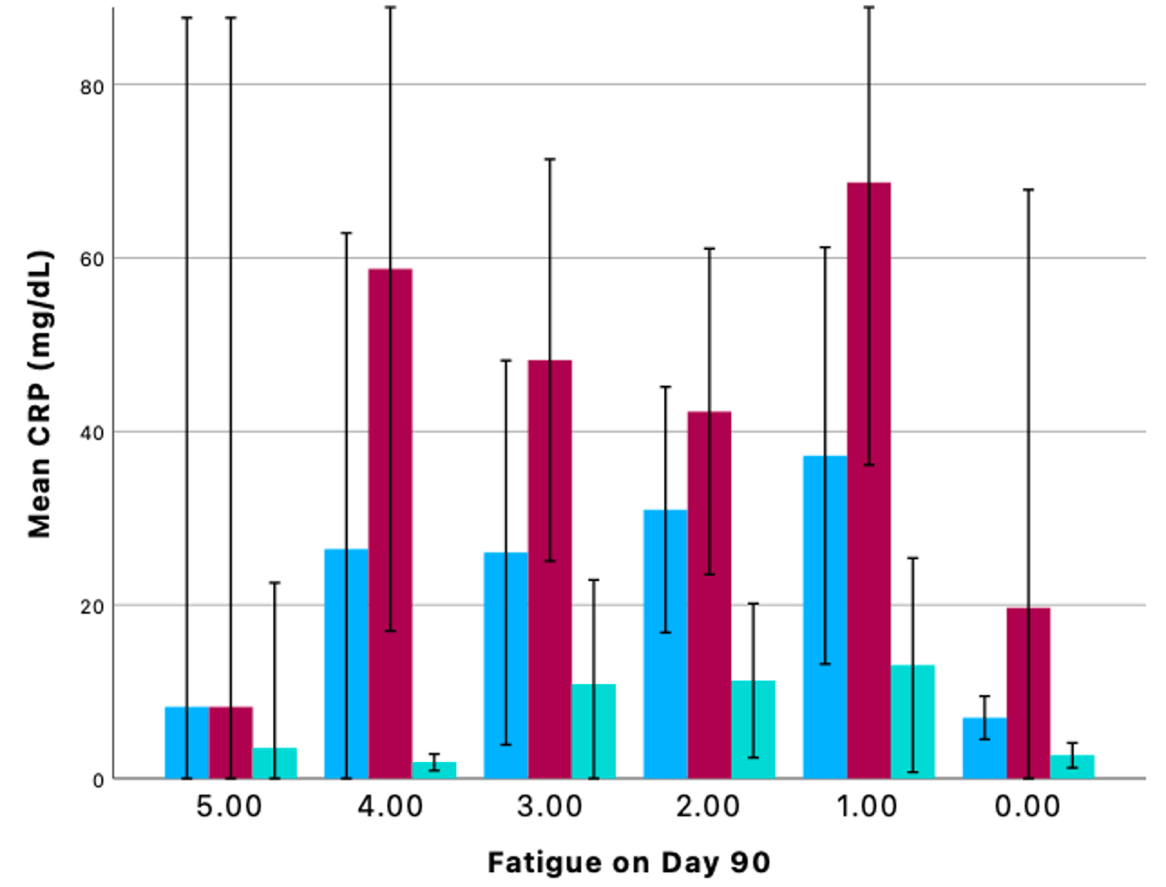
**SUPPLEMENTS**

Figure 4 - Mean CRP and fatigue on Day 90. Although there was significance between the unadjusted levels of fatigue at Day 90 and CRP at day 28 (turquoise), this trend loses significance after adjusting

*
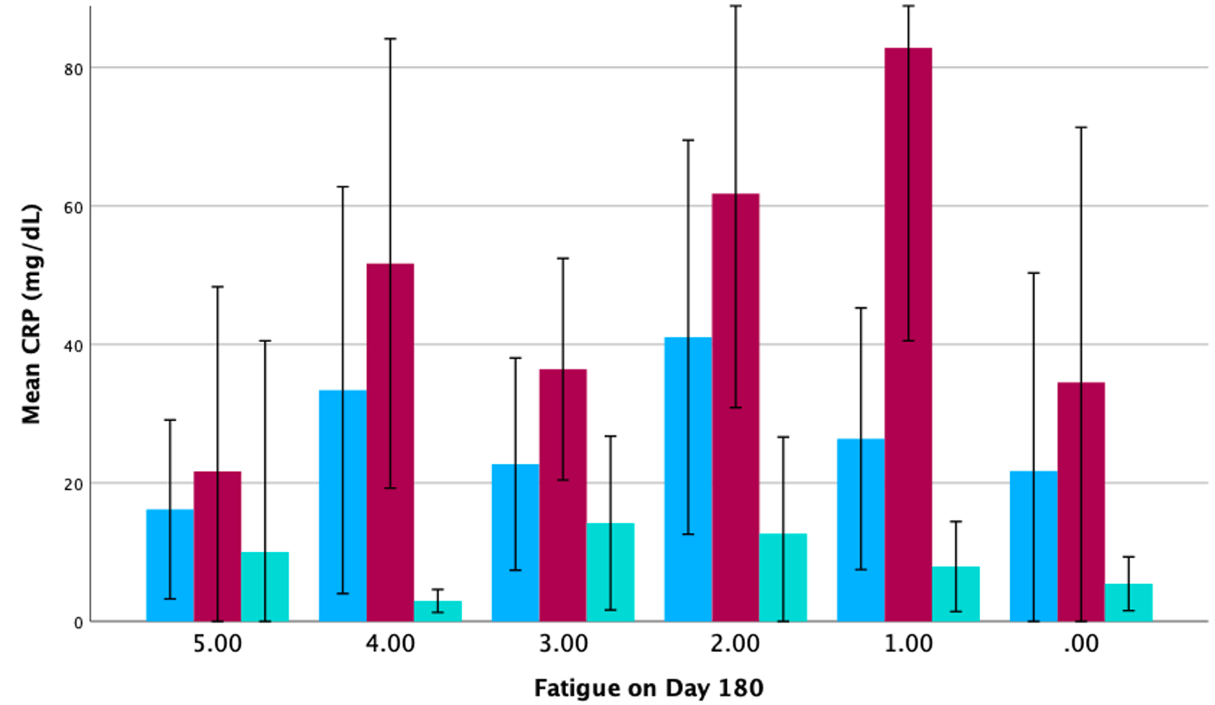
Figure 5 – Mean CRP and fatigue at day 180. There are no obvious trends between CRP and fatigue at this timepoint.*
